# Signal processing for RIS-assisted millimeter-wave/terahertz communications

**DOI:** 10.1093/nsr/nwad168

**Published:** 2023-06-05

**Authors:** Wei Wang, Wei Zhang

**Affiliations:** Peng Cheng Laboratory, China; School of Electrical Engineering and Telecommunications, The University of New South Wales, Sydney, Australia

## Abstract

The model-free and learning-based configuration of RIS is a new signal processing paradigm to overcome the challenges of embracing RIS in millimeter wave/Terahertz communications.

## INTRODUCTION

**Figure 1.**
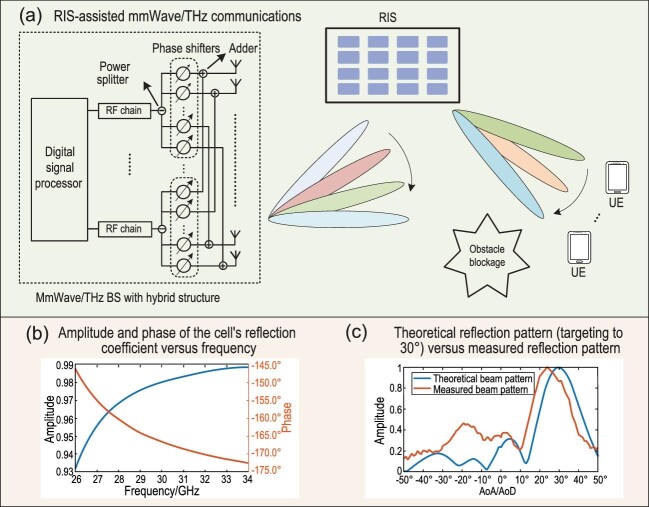
(a) Illustration of RIS-assisted mmWave/THz communications. (b) Amplitude and phase of the cell’s reflection coefficient versus frequency, showing the frequency-dependent mechanism of the RIS. (c) Theoretical reflection pattern (targeting to 30^○^) versus measured reflection pattern, where the theoretical reflection pattern is obtained by exhaustively searching for the best coding of a 12 × 12 RIS (the RIS’s reflecting element has four phase states, i.e. 2-bit phase quantization, and the reflecting elements in each column of the RIS share the same phase state).

The applications of millimeter-wave (mmWave, wavelength 1–10 mm) and terahertz (THz, wavelength 100 μm–1 mm) communications are facing the challenges of severe free-space path loss and obstacle blockage. Aided by directional beamforming, the free-space path loss of mmWave/THz communications with a fixed effective aperture is potentially capable of reaching the same level as the lower frequency band, rendering obstacle blockage the biggest hindrance to mmWave/THz communications. A reflecting intelligent surface (RIS) can play a vital role in mmWave/THz communications [1]. For one thing, the modulated metasurface offers a simpler low-cost architecture for mmWave/THz transceivers [1–3]. For another, the non-modulated metasurface is capable of assisting mmWave/THz communications by dynamically establishing on-demand coverage in dead zones where line-of-sight communication is inaccessible [1,4]. This paper investigates the signal processing challenges of the beam alignment process, which primarily attribute to the inability to sense signal and the imprecise reflection control of the RIS, for RIS-assisted mmWave/THz communications (Fig. 1(a)). We focus on the three candidate techniques for beam alignment in mmWave/THz communication, i.e. channel estimation, beam training and beamforming through learning, and discuss their suitability in RIS-assisted mmWave/THz communications.

## CHANNEL ESTIMATION

To compensate for RIS’s inability to sense signal, customized channel estimation techniques that require the coordination of the transmitter, the receiver and the RIS have been developed that accommodate both mmWave/THz communications and sub-6G communications [5]. However, channel estimation encounters several major challenges that degrade its applicability. First, the coordinated channel training process incurs excessive training overhead and reduces spectral efficiency. Second, the RIS is highly coupled with the radio access network, which increases the maintenance cost and reduces the scalability. Third, as the reflection response of the RIS changes with frequency [6], which is shown in Fig. 1(b), the phase shift of the RIS, which is regarded as part of the pilot signal, is inherently imprecise for the incident radio signal that is generally wideband.

In the mmWave/THz band, exploiting the sparse nature of the wireless channel and the directional transmission/reception of the mmWave/THz transceivers, beam training that acquires partial channel state information (CSI), i.e. the angle of arrival/departure (AoA/AoD) of the strongest path, is widely used to establish the data link. Similarly, in RIS-assisted mmWave/THz communications, beam training can be adopted to speed up the CSI (partial CSI) acquisition process.

## BEAM TRAINING

Beam training is the process of selecting the optimal passive beamforming vector through analyzing the wireless data collected from channel sounding/measurement. The reflective beam patterns of passive beamforming are usually directional and selected from a pre-defined codebook, e.g. discrete Fourier transform codebook and its variants, whereas the sensing patterns of the channel sounding can be either directional or non-directional. The beam-sweeping-based method and compressed-sensing- (CS) based method are two different strategies of beam training. Beam sweeping adopts the same codebook for both channel sounding and passive beamforming. It first measures the strength of the received signal and then selects the beam pattern that yields the highest signal strength. As beam sweeping is independent of knowledge about the RIS’s reflection response, it is robust to the imprecise phase control. CS-based beam training uses the non-directional random sensing pattern [7], the process of which includes random channel sensing and parameter estimation (e.g. AoA/AoD and path gain). Based on the estimated parameters, the closest beam pattern is chosen for passive beamforming. The CS-based method exploits the sparse nature of the mmWave/THz channel, and thus its training overhead is much less than beam sweeping. However, the performance of CS-based algorithms is degraded due to the imprecise phase control of the RIS that results in the non-negligible mismatch between the nominal and actual sensing matrices (Fig. 1(c)).

## BEAMFORMING THROUGH LEARNING

Recently, model-free designs that do not require the CSI of the RIS have been proposed for RIS configurations [8–10]. Model-free designs treat the RIS as a part of the wireless channel, rather than a component of the wireless communication system. The model-free design’s manner of inference is different from conventional beam training. Specifically, model-free designs derive the beam pattern directly from the channel measurements without drawing inference about the intermediate CSI, whereas beam training first draws inference from channel measurements to partial CSI and then from partial CSI to the beam pattern. Therefore, model-free designs of passive beamforming are data driven and can also be termed beamforming through learning. Beamforming through learning does not specify the beamforming codebook in advance. It directly adjusts the phase shift of each reflecting element based on the channel measurements. The algorithms of beamforming through learning include the majority-voting-based method (MVM) [8], the conditional sample-mean-based method (CSMM) [9], dither-based extremum seeking (DES)[10] and deep reinforcement learning (DRL) [10]. MVM and CSMM first collect a large batch of channel measurement data through random IRS configurations and then determine the optimal phase shift of each reflecting element by comparing the voting result or conditional sample mean. DES is an iterative algorithm that online updates the phase shift based on the latest channel measurement. DRL models the mmWave/THz transceiver pair, together with the channel between them, as the environment. Through the trial-and-error interaction with the environment, the RIS controller gradually learns the optimal policy to guide passive beamforming. Compared with beam sweeping and CS-based beam training, the flexibility of beamforming through learning in reflection pattern design renders it advantageous in coping with the more complex scenarios, e.g. the multi-user scenario and wideband scenario. A major concern of beamforming through learning is its convergence rate. *A priori* information has the potential to increase the convergence rate, e.g. starting point selection for DES, and *a priori* probability guided sampling for MVM and CSMM can contribute to the fast convergence of the algorithms. The underlying mathematical analysis regarding the convergence rate and the role of *a priori* information under different channel dynamics still needs more research efforts.

## FUTURE DIRECTIONS

In addition to beamforming through learning, auxiliary information-assisted designs are another technological pathway for fast and decoupled RIS configurations.

- The high frequency, large bandwidth and massive antenna array of mmWave/THz communications are inherently favorable conditions for positioning and sensing. Integrating positioning and sensing with mmWave/THz communications, the yielded position information and blockage information will significantly simplify the estimation of AoA/AoD.
- Visual information captured by high-definition cameras at the base station end can also be utilized to promote understanding of the radio environment.
- Multimodal deep learning that fuses auxiliary information with channel measurement information is a promising technology for fast and flexible RIS configurations.
